# Supplementary material for: Splice-Junction-Based Mapping of Alternative Isoforms in the Human Proteome
Source: Cell Rep. Author manuscript; Available in PMC 2020 Jan 15. (PMC6961840; doi:10.1016/j.celrep.2019.11.026)
Supplement: 2 [file NIHMS1546469-supplement-2.zip › DF1/PXD010154/Liver/Liver_68_ACADSB_ENSKMEKSVIQGLFQQGVGSFCLSEAGAGSDSFALK.pdf]

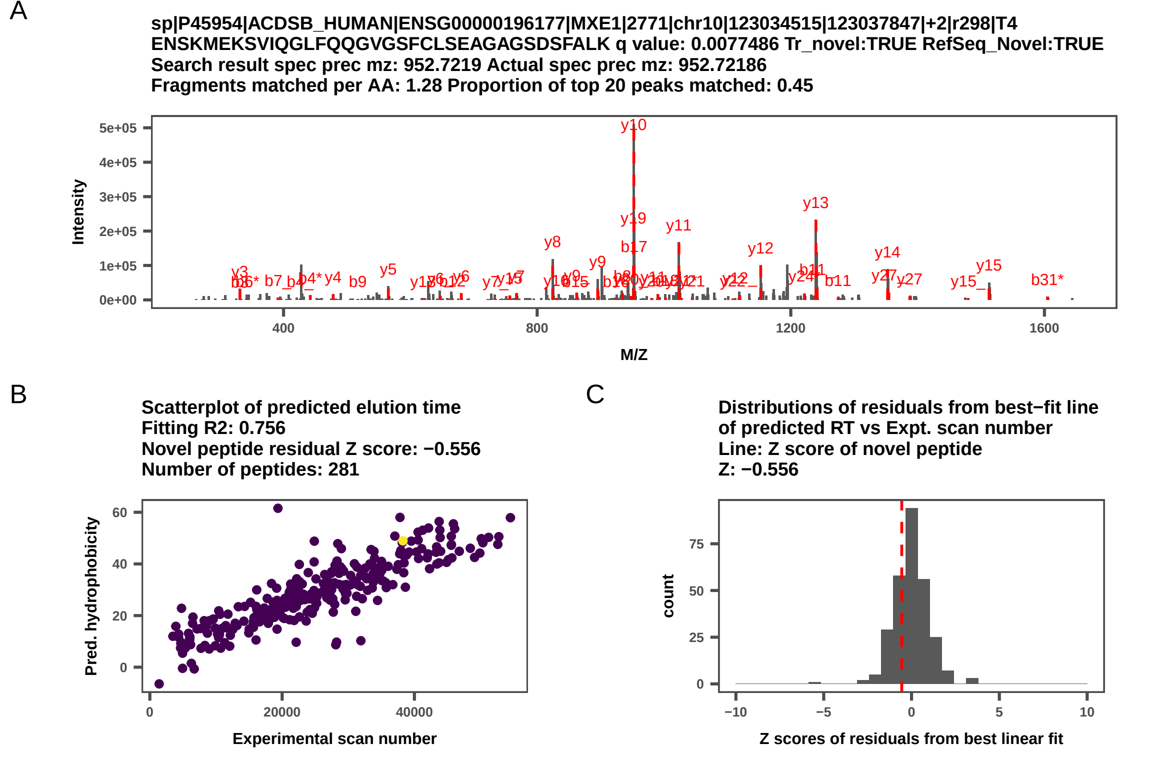
A
sp|P45954|ACDSB_HUMAN|ENSG00000196177|MXE1|2771|chr10|123034515|123037847|+2|r298|T4
ENSKMEKSVIQGLFQQGVGSFCLSEAGAGSDSFALK q value: 0.0077486 Tr_novel:TRUE RefSeq_Novel:TRUE
Search result spec prec mz: 952.7219 Actual spec prec mz: 952.72186
Fragments matched per AA: 1.28 Proportion of top 20 peaks matched: 0.45
Intensity
5e+05
4e+05
3e+05
2e+05
1e+05
0e+00
y10
y13
y19
y11
y8
b17
y12
y14
y9
y15
y5
y4
b9
b7_
y3
b11
y27
y15_
b31*
M/Z
400
800
1200
1600
B
Scatterplot of predicted elution time
Fitting R2: 0.756
Novel peptide residual Z score: −0.556
Number of peptides: 281
Pred. hydrophobicity
60
40
20
0
0
20000
40000
Experimental scan number
C
Distributions of residuals from best−fit line
of predicted RT vs Expt. scan number
Line: Z score of novel peptide
Z: −0.556
count
75
50
25
0
−10
−5
0
5
10
Z scores of residuals from best linear fit
